# Prevalence of Neutralizing Antibodies to Japanese Encephalitis Virus among High-Risk Age Groups in South Korea, 2010

**DOI:** 10.1371/journal.pone.0147841

**Published:** 2016-01-25

**Authors:** Eun Ju Lee, Go-Woon Cha, Young Ran Ju, Myung Guk Han, Won-Ja Lee, Young Eui Jeong

**Affiliations:** 1 Division of Arboviruses, National Institute of Health, Korea Centers for Disease Control and Prevention, Cheongju-si, Chungcheongbuk-do, Korea; 2 Japanese Encephalitis Regional Reference Laboratory for the WHO Western Pacific Region, Cheongju-si, Chungcheongbuk-do, Korea; 3 Department of Biomedical Sciences, Graduate School of Hallym University, Chuncheon-si, Gangwon-do, Korea; Ella Foundation, INDIA

## Abstract

After an extensive vaccination policy, Japanese encephalitis (JE) was nearly eliminated since the mid-1980s in South Korea. Vaccination in children shifted the affected age of JE patients from children to adults. However, an abrupt increase in JE cases occurred in 2010, and this trend has continued. The present study aimed to investigate the prevalence of neutralizing antibodies to the JE virus (JEV) among high-risk age groups (≥40 years) in South Korea. A plaque reduction neutralization test was conducted to evaluate the prevalence of neutralizing antibodies to JEV in 945 subjects within four age groups (30–39, 40–49, 50–59, and 60–69 years) in 10 provinces. Of the 945 enrolled subjects, 927 (98.1%) exhibited antibodies against JEV. No significant differences were found in the prevalence of neutralizing antibodies according to sex, age, or occupation. However, there were significant differences in the plaque reduction rate according to age and occupation; oldest age group had a higher reduction rate, and subjects who were employed in agriculture or forestry also had a higher value than the other occupations. We also found that three provinces (Gangwon, Jeonnam, and Gyeongnam) had a relatively lower plaque reduction rate than the other locations. In addition, enzyme-linked immunosorbent assays were conducted to determine recent viral infections and 12 (2.2%) subjects were found to have been recently infected by the virus. In conclusion, the present study clearly indicated that the prevalence of neutralizing antibodies has been maintained at very high levels among adult age groups owing to vaccination or natural infections, or both. In the future, serosurveillance should be conducted periodically using more representative samples to better understand the population-level immunity to JE in South Korea.

## Introduction

Japanese encephalitis (JE) is a highly prevalent human viral encephalitis in Asian countries. The causative pathogen, the JE virus (JEV), is a mosquito-borne flavivirus in the family *Flaviviridae* [1]. The JEV genome is a positive-sense and single-stranded RNA molecule with a length of 11 kb. The polyprotein consists of three structural proteins and seven non-structural proteins, and is flanked by un-translated regions at the 5' and 3' ends of the genome [1]. JEV has one serotype but it is genetically divided into five genotypes (I–V) based on the analysis of the envelope gene or complete genome sequences [2, 3].

Although the virus is transmitted by a zoonotic cycle between vector mosquitoes and pigs or water birds as amplifiers, humans and horses are infected incidentally and considered as dead-end hosts that cannot transmit the virus [4, 5]. Since the first recognized JEV infection in the 1870s in Japan, the affected areas expanded to most Asian countries in the 2010s [6, 7]. To date, outbreaks have been reported in over 20 countries located in temperate and tropical regions: Japan, China, Korea, Taiwan, Vietnam, Nepal, Pakistan, Bangladesh, India, Sri Lanka, Myanmar, Laos, Thailand, Cambodia, Malaysia, Indonesia, Philippines, Papua New Guinea, and the northern part of Australia. Despite the present availability of several vaccines, including inactivated or live-attenuated forms [8, 9], approximately 67,900 annual JE cases are estimated to occur in Asia and the western Pacific regions [6].

Japanese encephalitis is the sole autochthonous flavivirus infection in South Korea, although the tick-borne encephalitis virus has been isolated in nature [10], and imported flavivirus infections such as dengue, West Nile fever, and yellow fever have been reported annually [11, 12]. In South Korea, JE has been reported since the 1930s and is recognized as a significant threat to public health [13]. A large epidemic with several thousands of cases have been recorded every 2–3 years before the introduction of a mouse brain-derived inactivated vaccine from Japan in 1967 [14], which was administered to limited groups until the early 1980s. The vaccination program led to a dramatic decrease in the number of reported JE cases, from 12,055 cases with a mean annual incidence rate of 6.04 per 100,000 persons in 1961–1967 to 3,783 cases (mean incidence, 0.67) in 1968–1983 [11]. Following the last epidemic in 1982 (1,197 cases) and 1983 (139 cases), the Korean government started a mandatory vaccination of all children aged 3–15 years annually until 1994 [13]. Thereafter, the vaccination schedule has changed two times in 1995 and 2000. As a result, JE was considered a nearly eliminated disease, and only 55 cases (mean incidence, 0.004) were reported in 1984–2009 [11].

However, an abrupt increase in patients with JE occurred in 2010 (26 cases), and this trend is likely to continue [11]. From 2010 to 2014, 89 cases with JE (mean incidence, 0.04) were confirmed by laboratory testing. The health authority could not provide an explanation for the abrupt increase despite the careful analysis of data from the national JEV surveillance program [15]; compared with data from previous years, there was no increase in mosquito abundance or viral activity. Notably, the affected patients in 2010 were largely adults; 23 of the patients were older than 40 years, and the remaining three younger patients were not previously immunized [15]. Furthermore, of the 122 patients with JE confirmed between 2001 and 2014, 104 (85.2%) were older than 40 years (Table 1). A similar shift in the affected age of patients with JE was also reported in both Japan and Taiwan [16, 17]. This shift in age was mainly attributable to the long-term vaccination program for children.

**Table 1:** Age distribution among patients with Japanese encephalitis in South Korea between 2001 and 2014.

Year	2001	2002	2003	2004	2005	2006	2007	2008	2009	2010	2011	2012	2013	2014	Total (%)
No. of patients	1	6	1	0	6	0	7	6	6	26	3	20	14	26	122
No. of deaths	1	0	1	0	1	0	1	0		7	0	5	3	4	23 (18.9)
Incidence ^a^	0	0.01	0	0	0.01	0	0.01	0.01	0.01	0.05	0.01	0.04	0.03	0.05	
Age group (years)															
<10	0	0	0	0	0	0	0	0	0	0	0	2	0	0	2 (1.6)
10–19	0	1	0	0	0	0	0	0	0	1	0	0	0	1	3 (2.5)
20–29	0	2	0	0	0	0	0	0	1	1	0	0	0	2	6 (4.9)
30–39	0	1	0	0	1	0	1	0	1	1	1	1	0	0	7 (5.7)
40–49	0	2	0	0	2	0	3	4	3	9	1	5	3	5	37 (30.3)
50–59	1	0	0	0	0	0	2	1	1	9	1	9	5	8	37 (30.3)
≥ 60	0	0	1	0	3	0	1	1	0	5	0	3	6	10	30 (24.6)
Total	1	6	1	0	6	0	7	6	6	26	3	20	14	26	122

The raw data were collected from the Infectious Diseases Surveillance Yearbook [11], and were compiled by the author for this table.

^a^ Incidence rate per 100,000 persons; No., number.

It is important to monitor the immune status of the general population and implement appropriate measures, such as increasing the vaccine coverage rate or booster immunization for at-risk age groups. The health authority searched for information on the immunity levels to JE in adult age groups in South Korea. Although several studies on the vaccine efficacy or seroconversion before and after the summer season in children have been reported [14, 18–20], a nationwide investigation on the prevalence of neutralizing antibodies to JEV has not been well documented.

In this study, we collected serum samples from 945 subjects who represented the age and residence locations of South Korea and investigated the prevalence of neutralizing antibodies to JEV.

## Materials and Methods

### Ethics statement

The use of human sera in this study was approved by the Institutional Review Board of the Korea Centers for Disease Control and Prevention (IRB nos.: 2010-08CON-07-P on 20 January 2011, 2012-05CON-06-P on 10 July 2012, and 2013-01CON-06-P on 13 February 2013). Written informed consent was obtained from all the participants. The subjects’ names were not disclosed to the authors. The collected data included age, sex, residence location, occupation, and disease record (diabetes).

### Human serum samples

**Fig 1:**
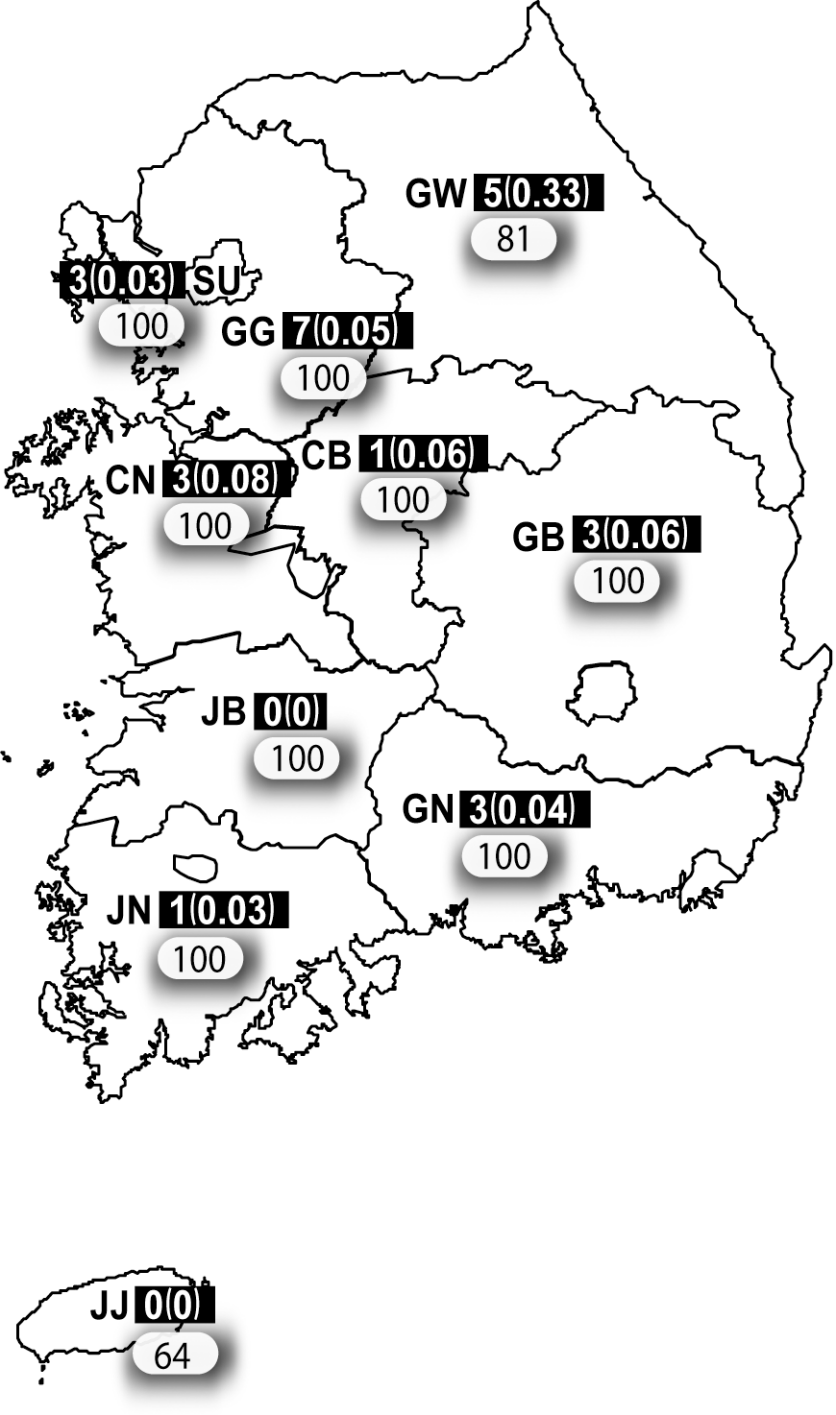
Study areas and distribution of study subjects. Human serum samples were obtained from the National Biobank of Korea. Using simple random sampling, 945 subjects were selected within four age groups from 10 provinces. The number of subjects per area is provided in the rounded rectangle. The number of patients with Japanese encephalitis in 2010 is provided in the black squares. In addition, the incidence rates per 100,000 persons in each province are provided in parenthesis. SU, Seoul; GG, Gyeonggi; CB, Chungbuk; CN, Chungnam; JB, Jeonbuk; JN, Jeonnam; GN, Gyeongnam; GB, Gyeongbuk; GW, Gangwon; JJ, Jeju.

We used serum samples that were collected during the Korea National Health and Nutrition Examination Survey (KNHANES) in 2010, which has been conducted annually since 1998 by the Korean Ministry of Health and Welfare, and it is a representative national survey consisting of a health questionnaire survey, health examination, and nutrition survey [21]. Multistage cluster sampling for the 2010 KNHANES resulted in 8,598 subjects representing each age group, sex, and residence location throughout the country. Of these, 5,894 individuals were of the target age (30–69 years) for this study, and 2,652 subjects (45.0%) agreed to deposit their serum samples in the National Biobank of Korea (Cheongju-si, Chuncheongbuk-do, Korea) for research purposes. Using simple random sampling methods, we selected a total sample of 945 subjects who were classified into four age groups from 10 provinces (Fig 1 and Table 2).

**Table 2:** Distribution of the subjects enrolled in this study.

		Number of sera by age group (birth year) ^a^				
Province	Sex	30–39 (1971–1980)	40–49 (1961–1970)	50–59 (1951–1960)	60–69 (1941–1950)	Total
Seoul	Female	13	12	13	12	50
(SU)	Male	12	13	12	13	50
Gyeonggi	Female	13	12	13	12	50
(GG)	Male	12	13	12	13	50
Gangwon	Female	13	12	13	12	50
(GW)	Male	9	5	7	10	31
Chungbuk	Female	13	12	14	13	52
(CB)	Male	13	9	13	13	48
Chungnam	Female	12	13	12	13	50
(CN)	Male	13	12	13	12	50
Jeonbuk	Female	15	13	13	14	55
(JB)	Male	7	14	11	13	45
Jeonnam	Female	10	16	14	16	56
(JN)	Male	9	10	14	11	44
Gyeongbuk	Female	12	13	12	13	50
(GB)	Male	13	12	13	12	50
Gyeongnam	Female	13	13	13	11	50
(GN)	Male	12	12	12	14	50
Jeju	Female	10	11	11	8	40
(JJ)	Male	5	10	4	5	24
Total		229	237	239	240	945

^a^ Age is defined based on the sample collection date in 2010.

Since the JE vaccination history was not available for each subject, we postulated it based on the vaccination policy in Korea. Subjects aged 30–39 years who were born between 1971 and 1980 should have been vaccinated at least once during their childhood (~15 years). Subjects aged 40–49 years who were born between 1961 and 1970 may have been vaccinated, but there was a limited quantity of the vaccine after its first introduction in 1967. Vaccinations in childhood would not have occurred in the subjects born before 1950 (aged 50–59 years and 60–69 years in this study) because the vaccine was unavailable. Therefore, together with the current age pattern of patients with JE, we defined those aged ≥40 years as the high-risk group for JE.

### Plaque reduction neutralization test

The plaque reduction neutralization test (PRNT) was performed according to the standard method of the Korea National Institute of Health, with modifications [22]. Briefly, BHK-21cells (ATCC, CCL-10) were grown in a six-well plate for 24–26 h, and the Nakayama strain of JEV was used to infect the cells. The human serum was heat-inactivated at 56°C for 30 min, diluted to 1:5 in 100 μL of minimum essential medium (MEM) supplemented with 10% fetal bovine serum (FBS) and penicillin/streptomycin antibiotics (Gibco, Grand Island, NY, USA). The serum was then mixed with equal volume of the virus that was titrated to 100 plaque forming unit (pfu) and incubated for 1 h at 37°C. Each virus/serum mixture (total volume, 200 μL) was inoculated onto the BHK-21 cell monolayer after draining the culture medium and allowing it to settle for 1 h at 37°C in a CO_2_ incubator. After removing the virus/serum mixture from the cell and briefly washing with phosphate buffered saline, 4 mL of pre-warmed overlay medium consisting of 0.9% Noble agar (Gibco), penicillin/streptomycin (100 units), and 4% FBS in MEM was poured onto each well. The cells were further incubated for 4–5 days in a CO_2_ incubator, and the overlay medium was removed. Each well was fixed with 10% formalin for 30 min and stained briefly with 1% crystal violet solution. Plate wells were washed with tap water and dried, and the plaques were counted. Each sample was tested in duplicate, and the mean values of the plaques were used to calculate the plaque reduction rates. Two plates of virus controls (12 wells) were included in each run, in which only the virus was infected with a titer of 100 pfu per well. If the mean plaque counts in each sample well were reduced by ≥50% compared with those from the virus-only wells, the sample was considered positive, with protective immunity against viral infection (World Health Organization guideline) [23].

The plaque numbers of the virus-only wells were used to check the assay qualification. We defined two criteria for qualified assay as follows. First, the mean plaque numbers of the virus-only wells should be between 50 and 150, as suggested in the literature [24]. Second, the coefficient of variation of plaque counts in the virus-only wells should be within 20% in each assay (this is an arbitrary qualification parameter of the authors’ laboratory).

### Enzyme-linked immunosorbent assay

To detect JEV-specific immunoglobulin (Ig) M antibodies in the serum samples, the JE-Dengue IgM Combo enzyme-linked immunosorbent assay (ELISA) (Panbio, Qld, Australia) and JE Detect IgM ELISA (InBios, Seattle, WA, USA) kits were used, according to the manufacturers’ instructions [25]. The JEV-specific IgG antibodies were detected using the JE Detect IgG ELISA kit (InBios) [26].

### Statistical analysis

All of the analyses were performed using PASW Statistics for Windows, version 18.0 (SPSS Inc., Chicago, IL, USA), and P values <0.05 were considered significant. Briefly, chi-square test was used to assess the difference between proportions, and the independent *t*-test and non-parametric Kruskal-Wallis test were used to analyze differences between the mean values.

## Results

### Prevalence of neutralizing antibodies to Japanese encephalitis virus

The overall prevalence of neutralizing antibodies to JEV was 98.1% (927/945) in those ≥30 years (Table 3). The prevalence was not significantly different between the sexes (P = 0.842) or age groups (P = 0.962). The prevalence according to the residence locations ranged from 93.0–100% (P <0.001). The prevalence in the Gangwon (95.1%), Jeonnam (93.0%), and Gyeongnam (96.0%) provinces was lower than that of the other provinces. The prevalence according to occupation (agriculture vs. other) was not significantly different (P = 0.210). We suspected that diabetes may lead to the negative antibody response. However, no one had a diabetic history among the 18 subjects who had a negative antibody response. In addition, the disease status did not affect the prevalence of the neutralizing antibody (P = 0.299).

**Table 3:** Prevalence of neutralizing antibodies to Japanese encephalitis virus.

Variable	Classification	No. of positive subjects/No. of tested	Positive rate (%)	P-value^a^
	Overall	927/945	98.1	
Sex				
	Female	493/503	98.0	0.842
	Male	434/442	98.2	
Age^a^ (years)				
	30–39 (1971–1980)	224/229	97.8	0.962
	40–49 (1961–1970)	232/237	97.9	
	50–59 (1951–1960)	235/239	98.3	
	60–69 (1941–1950)	236/240	98.3	
Province				
	Seoul	99/100	99.0	< 0.001
	Gyeonggi	100/100	100	
	Gangwon	77/81	95.1	
	Chungbuk	100/100	100	
	Chungnam	98/100	98.0	
	Jeonbuk	100/100	100	
	Jeonnam	93/100	93.0	
	Gyeongbuk	100/100	100	
	Gyeongnam	96/100	96.0	
	Jeju	64/64	100	
Occupation^b^				
	Agriculture or forestry	158/159	99.4	0.210
	Other	754/770	97.9	
Diabetes				
	Yes	55/55	100.0	0.299
	No	866/883	98.1	

^a^ Age is defined based on the sample collection date in 2010.

^b^ In the KNHANES in 2010, the occupation consists of seven classifications, except soldier. Other includes manager, clerks, service and sales, machine operator, simple labor, and no occupation (e.g., a housewife and student).

Since we performed PRNT at a single serum dilution factor, the end-point antibody titer for each serum was unavailable. Instead, we used the plaque reduction rate as the indicator of magnitude of the neutralizing antibody titer (Table 4). The mean plaque reduction rate was not significantly different according to sex (P = 0.117). As for the age group, the plaque reduction rate at 60–69 years was significantly higher than that of the other age groups (P <0.001). The plaque reduction rates according to the provinces were significantly different. Similar to the prevalence data, Gangwon (77.4%), Jeonnam (86.0%), and Gyeongnam (82.5%) provinces showed relatively low reduction rates than that of other provinces (P <0.001). Subjects who were employed in agriculture or forestry had a significantly higher plaque reduction rate (P = 0.011). The diabetes group had a higher plaque reduction rate (92.4%) than the normal group (88.0%).

**Table 4:** Plaque reduction rate to Japanese encephalitis virus.

Variable	Classification	No. of subjects	Mean plaque reduction rate (%)	SD	P-value
	Overall	945	88.2	13.8	-
Sex					
	Female	503	87.6	13.7	0.117
	Male	442	89.0	13.8	
Age^a^ (years)					
	30–39 (1971–1980)	229	87.2	14.1	<0.001
	40–49 (1961–1970)	237	85.3	13.5	
	50–59 (1951–1960)	239	88.6	13.6	
	60–69 (1941–1950)	240	91.7	13.8	
Province					
	Seoul	100	87.1	14.1	<0.001
	Gyeonggi	100	89.6	9.5	
	Gangwon	81	77.4	17.2	
	Chungbuk	100	92.3	10.0	
	Chungnam	100	87.7	15.8	
	Jeonbuk	100	94.1	7.0	
	Jeonnam	100	86.0	16.0	
	Gyeongbuk	100	94.9	6.3	
	Gyeongnam	100	82.5	16.8	
	Jeju	64	89.0	10.9	
Occupation ^b^					
	Agriculture or forestry	159	90.8	12.3	0.011
	Other	770	87.7	13.9	
Diabetes					
	Yes	55	92.4	11.7	0.022
	No	883	88.0	13.7	

^a^ Age is defined based on the sample collection date in 2010.

^b^ In the KNHANES in 2010, the occupation consists of seven classifications, except soldier. Other includes manager, clerks, service and sales, machine operator, simple labor, and no occupation (e.g., a housewife and student).

Eighteen subjects were negative for the neutralizing antibody in this study (Table 5). The mean age was 48.9 years (32–69 years) and the mean plaque reduction rate was 37.9% (0–49.5%). None of these 18 subjects had history of diabetes, and one person was employed in agriculture or forestry.

**Table 5:** Demographics of subjects with no neutralizing antibody to Japanese encephalitis virus.

No.	Province	Sex	Age (years)	Plaque reduction rate (%)	Occupation^a^
1	Gangwon	Female	35	29.7	Other
2		Male	52	38.8	Other
3		Female	57	32.2	Other
4		Male	62	25.6	Other
5	Gyeongnam	Female	35	34.4	Other
6		Male	45	48.4	Agriculture or forestry
7		Female	49	33.5	Other
8		Female	69	43.3	Other
9	Jeonnam	Female	32	45.6	Other
10		Female	36	43.3	Other
11		Male	39	46.4	Other
12		Female	44	47.9	Other
13		Male	46	39.9	Other
14		Male	47	41.2	Other
15		Male	50	40.7	Other
16	Chungnam	Female	53	41.3	Other
17		Female	65	49.5	Other
18	Seoul	Male	64	0	Other

^a^ In the KNHANES in 2010, occupation consists of seven classifications, except soldier. Other includes manager, clerks, service and sales, machine operator, simple labor, and no occupation (e.g., housewife and student).

### Recent infection to Japanese encephalitis virus

Among the 945 subjects enrolled in this study, 12 (2.2%, 95% confidence interval: 1.2–3.8%) were IgM-positive for at least one of the two ELISA kits, which indicated a recent viral infection (Table 6). Positive results were obtained from five of the 10 provinces, where sampling was done between January and October 2010. All subjects had ≥90% plaque reduction rate with the PRNT.

**Table 6:** Evidence of recent infection by Japanese encephalitis virus (N = 549).

No.	Province	Age group	Collection date	IgM ELISA^a^		IgG ELISA
				InBios	Panbio	InBios
1	Seoul	50–59	Jan 21, 2010	equiv	+	-
2	Gyeonggi	30–39	Oct 07, 2010	+	+	-
3		40–49	Oct 21, 2010	equiv	+	-
4		40–49	Feb 24, 2010	+	+	-
5		50–59	May 27, 2010	+	+	equiv
6	Jeonbuk	60–69	May 18, 2010	+	+	-
7	Chungnam	40–49	Aug 12, 2010	+	-	equiv
8		50–59	Jun 14, 2010	+	+	-
9		60–69	Mar 18, 2010	equiv	+	equiv
10		60–69	Mar 18, 2010	+	-	-
11	Jeju	40–49	Apr 26, 2010	+	+	equiv
12		50–59	Jan 19, 2010	+	+	+

^a^ Two brands of ELISA kits were used: InBios (Seattle, WA, USA) and Panbio (Panbio, Qld, Australia). equiv, equivocal; +, positive; -, negative.

## Discussion

To our knowledge, this is the first nationwide surveillance that investigated the prevalence of the JE neutralizing antibody among the general population in South Korea. It was initiated based on an urgent need to respond to an increase in JE cases in 2010. Since it is very important to select subjects who can represent the target age and residence locations throughout the country, we decided to use serum samples collected by the KNHANES in which all subjects were selected by a statistically robust sampling strategy by the Korean government. In 2010, only 2,652 of the 8,894 subjects of the KNHANES were available for sampling because many subjects did not agree to deposit their serum samples. We finally selected 945 subjects from 10 provinces, and divided them into four age groups. The limited sample size could have skewed the data. Thus, the results obtained from this study should be interpreted with caution.

The overall prevalence of neutralizing antibodies to JEV was very high, with a positive rate of 98.1% (927/945). Interestingly, no age-specific difference in the prevalence was found, which indicates no differences between the vaccinated and unvaccinated age groups. These results differ from those reported in Japan and Taiwan where the vaccination policy spanned 40 years and a shift in affected ages was also reported [17, 23]. In both of these countries, a prevalence of neutralizing antibodies of <50% or a relatively low prevalence was detected in at least one age group: those 30–59 years in Japan in 2004 and those 27–39 years in Taiwan in 2002. The high prevalence in Korea may be explained by the comparative magnitude of the JE epidemics and extensive vaccination program. Following the largest epidemic of approximately 2,000 cases in 1965 in Japan, the annual number of cases decreased to several hundred by the late 1960s, and only a small number of cases (2–54 patients) have been reported annually since 1982 [17]. In Taiwan, a maximum of 200–300 cases were reported in 1966–1974, and an average of 20–30 cases have been reported annually since implementing a vaccine policy in the late 1960s [27]. In Korea, three large epidemics with 1,000–3,500 cases were reported in 1961–1968, and the latest epidemic in 1983 involved 139 reported cases [13]. The repeated and large JE epidemics within a short period may have led to the long-term maintenance of neutralizing antibodies in a high percentage of the general population in Korea. Generally, natural infections elicit greater and longer-lasting immune responses compared with vaccine-induced immunity [28, 29]. Consistent with this assumption, the unvaccinated age group (60–69 years) had a higher plaque reduction rate than the younger vaccinated age group in this study. In addition, the extensive vaccination schedule initiated in the early 1980s could have contributed to the high positive rates of neutralizing antibodies in the Korean population; this involved a three-dose primary vaccination from 3 years and annual boosters until 15 years [13]. The vaccine coverage rate of children in Korea was maintained at >90% from 1985 to the mid-1990s. Currently, the vaccination schedule is a three-dose primary course at 1–3 years of age and two booster immunizations at ages 6 and 12. A recent survey conducted in 2008 indicated that the vaccine coverage rate in children had decreased to 80% for the first and second doses and 53.0% for the third dose [30]. However, the sample sizes were small (n = 585 for the first and second doses and n = 440 for the third dose), potentially resulting in under-estimation of the rates. In South Korea, all parents should submit immunization certificates for their children to the school authority before starting elementary school. However, as the length of time from the last immunization increases, more people in this age group are likely to be infected with JEV due to the waning of neutralizing antibodies.

Both the neutralizing antibody prevalence and magnitude of the antibody titer (inferred by the plaque reduction rate) were significantly different according to the residence locations. In particular, the Gangwon province showed a lower antibody prevalence of 95.1% and a plaque reduction rate of 77.4%. We were unable to obtain specific information on the vaccine coverage rate for this province. Compared with other provinces, except Seoul (a metropolitan city), the rice cultivation area (38,809 ha) in the Gangwon province was the second smallest, and the number of pig farms (293) was the smallest as of 2010. Thus, fewer opportunities for exposure to the virus in this province may have caused this result. Indeed, in 2010, the incidence rate of 0.33 in Gangwon province was the highest among the 10 provinces (average incidence rate, 0.05; range, 0–0.33). Although the Jeonnam and Gyeongnam provinces also showed a low plaque reduction rate than the average value (88.2%) in this study, the incidence rates were not higher than that of other provinces. These findings suggest that more complicated factors may be involved in the disease occurrence. Interestingly, the subjects’ occupation caused a meaningful difference in the plaque reduction rate, which is consistent with the general assumption that agriculture (or forestry) would be associated with a higher chance for virus infection than other occupations.

Recent infections were detected in 12 (2.2%) of 945 subjects using commercially available ELISA kits; nine of the sera were collected between January and June in five provinces. JEV is normally detected between July and October in South Korea, and patients are reported in September to early December [13, 15, 31]. Therefore, these nine cases were likely to be infected in the previous year. The antibodies elicited by JEV can be detected by ELISA for months and up to a year later [26, 32]. Negative results of the IgG ELISA in all 12 cases were attributed to the intrinsic low detection efficacy of the kit, which has been described previously [22, 26]. In Japan, the annual infection rate of 2.6% was estimated from a survey using the ELISA kits in the Kumamoto and Tokyo provinces between 2004 and 2008 (n = 345) [33]. Therefore, IgM ELISA kits may be useful for periodically monitoring recent infections in the general population. It is important to investigate the viral infection rates at a given time to assist health authorities in estimating the extent of viral activity more precisely and in determining annual changes.

The high prevalence of neutralizing antibodies to JE may be helpful for antiviral therapy for JE. A neutralizing antibody inhibits virus replication and virus spread during the initial stage in patients with encephalitis [34]. Recently, immunotherapy of virus infections using high titer antibodies has been documented [35, 36]. Clinical evidence supports that intravenous immunoglobulin containing JEV antibodies have a positive effect on treating patients with JE [37, 38]. A similar treatment effect was also reported in patients with West Nile virus infection [39].

Despite the limited sample size, it is clear that the prevalence of neutralizing antibodies to JEV were maintained at a high level regardless of the age groups in South Korea. Undoubtedly, vaccination and/or natural infection leads to this phenomenon. The health authorities need to maintain higher vaccine coverage rates and conduct effective vector control measures during the virus transmission season.

## Conclusions

The present study indicates that the prevalence of neutralizing antibodies to JEV has been maintained at very high levels among the general population in South Korea. As this study is the first nationwide surveillance on the prevalence of the JE neutralizing antibody, the data will be useful for the health authority to understand the current immune status of the general population and make an appropriate policy to control JE. Further surveillance should be conducted periodically using more representative samples to better understand the population-level immunity to JEV.

## Supporting Information

S1 Table Quality assessment of the plaque reduction neutralization test. (PDF)

S2 Table Rice cultivation area and the scale of pig farms in Korea in 2010. (PDF)
