# Correction: Prevalence of Neutralizing Antibodies to Japanese Encephalitis Virus among High-Risk Age Groups in South Korea, 2010

**DOI:** 10.1371/journal.pone.0151227

**Published:** 2016-03-07

**Authors:** Eun Ju Lee, Go-Woon Cha, Young Ran Ju, Myung Guk Han, Won-Ja Lee, Young Eui Jeong

The caption for Table 6 is incorrect. Please see the corrected Table 6 here.

**Table 6:** Evidence of recent infection by Japanese encephalitis virus (N = 945)

No.	Province	Age group	Collection date	IgM ELISA^a^		IgG ELISA
				InBios	Panbio	InBios
1	Seoul	50–59	Jan 21, 2010	equiv	+	-
2	Gyeonggi	30–39	Oct 07, 2010	+	+	-
3		40–49	Oct 21, 2010	equiv	+	-
4		40–49	Feb 24, 2010	+	+	-
5		50–59	May 27, 2010	+	+	equiv
6	Jeonbuk	60–69	May 18, 2010	+	+	-
7	Chungnam	40–49	Aug 12, 2010	+	-	equiv
8		50–59	Jun 14, 2010	+	+	-
9		60–69	Mar 18, 2010	equiv	+	equiv
10		60–69	Mar 18, 2010	+	-	-
11	Jeju	40–49	Apr 26, 2010	+	+	equiv
12		50–59	Jan 19, 2010	+	+	+

^a^ Two brands of ELISA kits were used: InBios (Seattle, WA, USA) and Panbio (Panbio, Qld, Australia). equiv, equivocal; +, positive; -, negative.

There is an error in the tenth sentence of the Abstract. The correct sentence is: In addition, enzyme-linked immunosorbent assays were conducted to determine recent viral infections and 12 (1.3%) subjects were found to have been recently infected by the virus.

There is an error in the first sentence of the “Recent infection to Japanese encephalitis virus” section of the Results. The correct sentence is: Among the 945 subjects enrolled in this study, 12 (1.3%, 95% confidence interval: 0.7–2.2%) were IgM-positive for at least one of the two ELISA kits, which indicated a recent viral infection (Table 6).

There is an error in the first sentence of the fourth paragraph of the Discussion. The correct sentence is: Recent infections were detected in 12 (1.3%) of 945 subjects using commercially available ELISA kits; nine of the sera were collected between January and June in five provinces.
