# Supplementary material for: Prevalence of Neutralizing Antibodies to Japanese Encephalitis Virus among High-Risk Age Groups in South Korea, 2010
Source: PLoS One. 2016 Jan 25;11(1):e0147841. doi: 10.1371/journal.pone.0147841 (PMC4725746; doi:10.1371/journal.pone.0147841)
Supplement: S1 Table — (PDF) [file pone.0147841.s001.pdf]

S1_Table. Quality assessment of the plaque reduction neutralization test

Experimenter	Mean no. of plaques in control wells (range)	Mean CV (range)	Mean no. of control wells readable (range)	No. of runs
A	101.9 (60.5–138.8)	8.7 (3.4–15.4)	10.3 (6–12)	25
B	97.7 (67.0–118.1)	8.7 (3.4–11.4)	10.2 (8–12)	13

For each experiment, the plaques of the control wells were counted and examined to determine if they met the criteria described in the Methods section. CV: coefficient of variation.

Description: Of the total 43 assays conducted, five runs failed to meet the criteria for quality acceptance, and the remaining 38 runs were used for quality assessment and to compare the performance between experimenters. The two experimenters showed good performance, with minimum differences (Table 2). On average, 10 (83.3%) of the 12 virus control wells were countable in each assay. The overall plaque numbers in the virus control wells were 60.5–138.8, and the largest CV was 15.4%. The mean plaque numbers of the control wells were not significantly different between the experimenters (unequal variance *t*-test, P= 0.552).
