# Supplementary material for: Prevalence of Neutralizing Antibodies to Japanese Encephalitis Virus among High-Risk Age Groups in South Korea, 2010
Source: PLoS One. 2016 Jan 25;11(1):e0147841. doi: 10.1371/journal.pone.0147841 (PMC4725746; doi:10.1371/journal.pone.0147841)
Supplement: S2 Table — (PDF) [file pone.0147841.s002.pdf]

S2 Table. Rice cultivation area and the scale of pig farms in Korea in 2010

Province	Rice cultivation area (ha)	No. of pig farms
Seoul	267	1
Gyeonggi	95,894	1,213
Gangwon	38,809	293
Chungbuk	46,758	403
Chungnam	156,456	1,339
Jeonbuk	134,123	833
Jeonnam	180,163	1,257
Gyeongbuk	116,472	802
Gyeongnam	83,950	1,078
Jeju	24	330
Total	852,916	7,549

The raw data were collected from the Korean Statistical Information Service (http://kosis.kr).
